# Choroidal vascularity index change in macular telangiectasia type 2

**DOI:** 10.1371/journal.pone.0262112

**Published:** 2022-04-07

**Authors:** Heejeong Chun, Hyun Suh, Joo Young Kim, Jae Hyuck Kwak, Rae Young Kim, Mirinae Kim, Young-Gun Park, Young-Hoon Park

**Affiliations:** 1 Department of Ophthalmology and Visual Science, Seoul St. Mary’s Hospital, College of Medicine, The Catholic University of Korea, Seoul, Korea; 2 Catholic Institute for Visual Science, College of Medicine, The Catholic University of Korea, Seoul, Korea; Massachusetts Eye & Ear Infirmary, Harvard Medical School, UNITED STATES

## Abstract

**Purpose:**

To analyze choroidal structure using subfoveal choroidal thickness (SFCT) and choroidal vascularity index (CVI) in Macular Telangiectasia (MacTel) type 2.

**Methods:**

Medical records of 43 eyes with MacTel type 2 and 30 sex and age-matched healthy eyes were retrospectively reviewed. Their SFCT and CVI were measured using the SS-OCT scan passing through the central fovea and image binarization. The difference in baseline SFCT and CVI from each group and their yearly changes up to second year of follow up were analyzed. The baseline characteristics of the groups were also compared.

**Results:**

The baseline characteristics, including CVI and SFCT, of the MacTel group and the control group were not significantly different, except for BCVA. The mean CVI of MacTel group were 64.59 ± 2.92%, 63.76 ± 2.67%, and 62.97 ± 2.74% (*p* < 0.001) whereas that of control group were 63.33 ± 2.45%, 63.04 ± 2.46%, and 63.43 ± 2.25% (*p* = 0.636) at baseline, 1 and 2 years, respectively. The mean SFCT of MacTel group were 324.65 ± 89.65μm, 326.14 ± 93.11μm, and 322.65 ± 91.77μm (*p* = 0.436), whereas that of control group were 304.30 ± 51.86 μm, 300.86 ± 52.64μm, and 298.55 ± 53.71μm (*p* = 0.275) at baseline, 1 and 2 years, respectively.

**Conclusion:**

CVI decreases at a faster rate in MacTel type 2 in comparison with healthy subjects. This may suggest possible choroidal involvement in the progression of MacTel type 2.

## Introduction

Macular telangiectasia (MacTel) type 2 is known as a bilateral disease that involves changes of the macular capillary network and neurosensory atrophy [1]. Its characteristic telangiectasia and dilation of the retinal capillaries occur mainly in the juxtafoveal area, especially temporally to the fovea [2]. Its macular alterations include various clinical findings, such as lack of foveolar reflex, reduced retinal transparency, crystalline deposits, ectatic capillaries, blunted/dilated retinal vessels, foveal atrophy, pigment-hypertrophy, lamellar or full thickness macular hole, and neovascular complex [1]. Symptoms are known to start in the fifth or sixth decade of life, and the disease may be misdiagnosed as age-related macular degeneration when present with neovascularization [1,3].

MacTel was classified into 3 groups and each group into A and B by Gass and Blondi [4]. Group 1 is a unilateral disease commonly diagnosed as congenital in young men and assumed as a variant of Coats’ disease, whereas group 2 is a bilateral disease affecting middle-aged and elderly patients and characterized by macular capillary changes, foveal cavitations and loss of outer retinal structures. Group 3 is a rare bilateral disease with occlusive vasculopathy affecting the macula, which is often associated with systemic diseases. As for the subgroups, group 1A and 1B involve juxtafoveolar telangiectasis area of >2 clock hours and ≤2 clock hours, respectively. The main difference of group 2B compared with group 2A is that it is juvenile and familial. Group 3B involves additional CNS-vasculopathy. In addition, group 2A, the most common type of the disease, was subdivided into 5 stages. Main characteristics of each stage were as follows: stage 1) diffuse hyperfluorescence in late phase fluorescein angiography (FA); stage 2) reduced parafoveolar retinal transparency; stage 3) dilated right angled venules; stage 4) intraretinal pigment clumping; stage 5) vascular membranes [1]. Later, Yannuzzi et al. [5] simplified this classification into Type 1 (Aneurysmal telangiectasis, Gass group 1) and Type 2 (Perifoveal telangiectasis, Gass group 2A), and categorized group 2A stages 1–4 as a non-proliferative phase and stage 5 as a proliferative phase with subretinal neovascular complex.

Even though the pathogenesis of MacTel type 2 is still under investigation, previous studies showed that Müller cell dysfunction or alteration is greatly responsible for the disease progress [6,7]. Several studies have discussed that the choroid may also be involved in the disease process as retinal-choroidal anastomoses are observed, suggesting it can be more than just a neurodegenerative disease with vascular abnormalities [8,9]. However, studies on the choroidal changes in MacTel type 2 have not yet had a consensus on the result, for example, one study showed an increase in choroidal thickness(CT) and others showed no difference in the disease compared with controls [10–12]. Also, previous study analyzed choroidal vessel density(CVD) by binarizing *en face* swept-source optical coherence tomography(SS-OCT) images but did not show a significant difference in MacTel type 2 compared with controls [10].

In order to acquire a more accurate assessment on changes in choroidal vascularity, recent studies are now analyzing the choroidal vascularity index (CVI), which is the ratio of the vascular luminal area (LA) to the total choroidal area (TCA) of choroid [13–16]. CVI is similar to CVD in a way that they both measure the percentage area occupied by the choroidal vessels. The difference is that CVI calculates the vascular area using the SS-OCT scan passing through the central fovea whereas CVD uses *en face* OCT images. Through many studies, it is suggested that when compared to choroidal thickness, CVI has a lesser variability and is influenced by fewer physiologic factors [17]. Therefore, CVI could be a better parameter for evaluating the choroidal vasculature. And to our knowledge, CVI has not yet been evaluated in MacTel type 2.

The purpose of this study was to confirm if there is any significant difference in choroid structure and vascularity by analyzing CT and CVI in MacTel type 2 compared with healthy controls, and to evaluate their changes over time.

## Materials and methods

### Study population

We retrospectively reviewed the medical records of 43 eyes with MacTel type 2 and 30 sex and age-matched healthy eyes whose first visit to our clinic was from January 2017 to January 2019 at Seoul St. Mary’s Hospital in Korea. This retrospective study was conducted in accordance with the tenets of the Declaration of Helsinki, and all protocols were approved by the institutional review board of The Catholic University of Korea. The institutional review board of The Catholic University of Korea waived the need of informed consent due to the retrospective nature of the study.

Eyes diagnosed with MacTel type 2, except for stage 5, based on the Gass classification [4] were included in the study. The exclusion criteria were as follows: 1) Eyes with any previous vitreomacular surgery, cataract surgery in the preceding 1 year, laser treatment, or ocular trauma, 2) high myopia with refractive errors of more than ± 6 diopters (as spherical equivalent), 3) systemic diseases that could affect the eye, except for diabetes and hypertension, 4) presence of other ocular diseases, including diabetic retinopathy, glaucoma, age-related macular degeneration, retinal vein occlusion, neurodegenerative disease or pachychoroidal pigment epitheliopathy, 5) media opacity disrupting image quality, 6) patients who did not meet their follow up schedules.

Medical and ophthalmologic history were recorded and ocular examinations, including best-corrected visual acuity (BCVA), refraction, non-contact pneumatic tonometry, slit-lamp biomicroscopy, and dilated fundus examination were done at each visit to the clinic.

### Choroidal imaging

OCT images were acquired at each visit and the images from the initial visit (baseline) and after 1 and 2 years during follow up were used in the study. Due to the retrospective nature of the study, the time at which these images were taken could not be controlled. OCT imaging was performed with a swept source (SS)-OCT device (DRI Triton, Topcon, Tokyo, Japan) using a 1050-nm wavelength light source, and a scanning speed of 100,000 A-scans/s. A 6-line radial pattern scan (1,024 A-scans) centered on the fovea was obtained from each eye.

Subfoveal choroidal thickness (SFCT) was obtained by manually measuring the distance between the outer border of the retinal pigment epithelium and the inner border of the suprachoroidal space at the foveal center using digital calipers provided by the OCT software.

All assessments of OCT images were conducted by two independent retina specialists (R.Y.K. and M.K.) who were blinded to the patient’s other imaging findings and clinical histories.

### Image binarization

**Fig 1:**
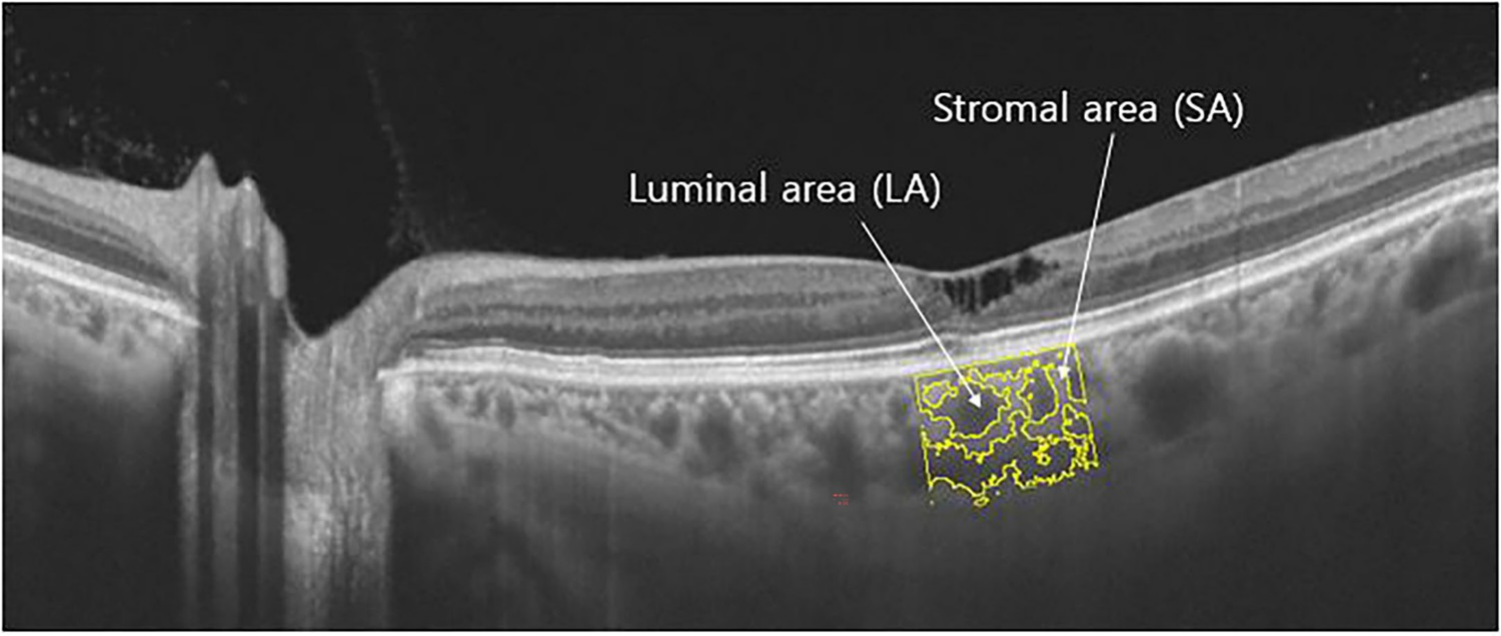
Representative swept source OCT image of Mactel type 2 eye to measure the CVI. The subfoveal region of 1,500μm was separated to the luminal area (LA) and the stromal area (SA).

CVI was measured using the SS-OCT scan passing through the central fovea, and the scan was segmented using the protocol described by Agrawal et al. [13] Image binarization was done using Image J software (Version 1.51; https://imagej.nih.gov/ij/). The polygon selection tool was used to select the total choroidal area (TCA) under the subfoveal region of 1500 μm (750 μm either side of the fovea), and regions of interest (ROIs) were added to the ROI manager. The image was then converted into 8 bit and separated to the luminal or vascular area (LA) and the stromal area (SA) by applying a Niblack auto-local threshold tool. Next, the image was converted back to an RGB (red, green, blue) image, and the LA was determined by applying the color threshold tool and added to the ROI manager. Then the LA within the initially selected polygon was measured by selecting and combining both areas previously added in the ROI manager. The CVI is then measured by calculating the ratio of LA to TCA. The representative swept source OCT image of Mactel type 2 eye to measure the CVI was shown in Fig 1.

### Statistical analysis

The statistical analyses were performed using the Statistical Package for the Social Sciences for Windows ver. 22.0 (SPSS Inc., Chicago, IL, USA). Shaprio-Wilks test was used to confirm a normal distribution of the continuous variables, and parametric tests were applied for comparison. The baseline characteristics of MacTel group and control group were compared using independent t-test. Then we analyzed the changes of CVI and SFCT in MacTel group and control group at baseline, 1 and 2 years during follow up by applying repeated measures ANOVA tests and post hoc analyses. The data were expressed as mean with standard deviation and *p* values < 0.05 were considered statistically significant.

## Results

This study included 43 eyes with MacTel type 2 and 30 sex and age-matched control eyes without any ocular abnormalities. Demographic and ophthalmic characteristics of the study participants are summarized in Table 1. The mean age of the MacTel patients and the control patients were 63.86 ± 6.94 years and 65.47 ± 6.96 years, respectively. Out of 43 MacTel eyes, 14 eyes were from male patients, 32 eyes were with diabetes and 21 eyes were with hypertension. The mean refractive errors were -0.10 ± 2.19 diopters and the mean BCVA were 0.17 ± 0.14 logMAR (20/30 Snellen equivalent) in the MacTel group at initial visit. The MacTel group had the mean intraocular pressure of 14.12 ± 3.58 mmHg, the mean SFCT of 324.65 ± 89.65 μm, and the CVI of 64.59 ± 2.92%. These baseline measurements from the MacTel group were not significantly different from the baseline measurements from the control group, except for BCVA.

**Table 1:** Demographic and clinical characteristics of the study participants at baseline.

	Total (n = 73)	MacTel (n = 43)	Control (n = 30)		*p*-value
Age, years	64.52 ± 6.95	63.86 ± 6.94		65.47 ± 6.96	0.335
Gender, male	25	14		11	0.720
Diabetes	54	32		22	0.919
Hypertension	42	21		21	0.070
SE, diopter	-0.10 ± 2.01	-0.10 ± 2.19		-0.11 ± 1.82	0.982
BCVA, logMAR (Snellen)	0.12 ± 0.13 (20/26)	0.17 ± 0.14 (20/30)		0.04 ± 0.05 (20/22)	**<0.001**
IOP, mmHg	14.21 ± 3.33	14.12 ± 3.58		14.33 ± 2.99	0.786
SFCT, μm	316.29 ± 76.63	324.65 ± 89.65		304.30 ± 51.86	0.225
CVI, %	64.07 ± 2.79	64.59 ± 2.92		63.33 ± 2.45	0.057

Data are expressed as mean ± standard deviation (95% confidence interval). Independent t-tests.

Abbreviations: BCVA, best-corrected visual acuity; CVI, Choroidal vascularity index; IOP, intraocular pressure; logMAR, logarithm of the minimum angle of resolution; MacTel, macular telangiectasia; SE, spherical equivalent; SFCT, subfoveal choroidal thickness.

**Fig 2:**
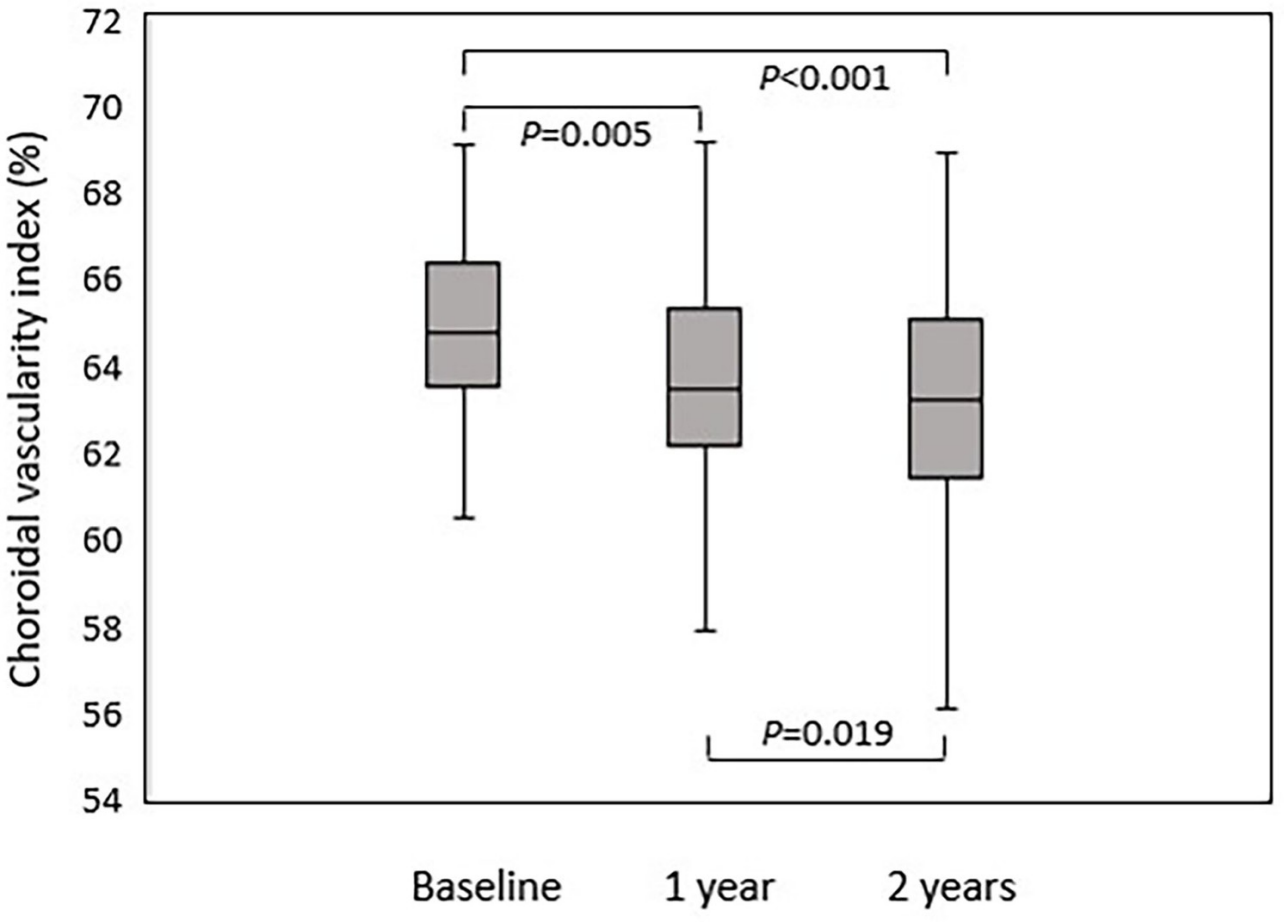
Choroidal vascularity index in MacTel group evaluated by repeated measures ANOVA tests and post hoc analysis. Reduction in choroidal vascularity index was statistically significant between baseline and 1 year(*p* = 0.005), 1 year and 2 years(*p* = 0.019), and baseline and 2 years(*p*<0.001).

The CVI and SFCT changes of each group were observed on a yearly basis, which is summarized in Table 2. The mean CVI of MacTel group were 64.59 ± 2.92%, 63.76 ± 2.67%, and 62.97 ± 2.74% at baseline, 1 and 2 years, respectively. In this group, the repeated measures ANOVA test showed *p* < 0.001, and the *p* value of post hoc analysis for baseline versus 1 year was 0.005, for 1 year versus 2 years was 0.019, and for baseline versus 2 years was <0.001 (Fig 2). The mean CVI of control group were 63.33 ± 2.45%, 63.04 ± 2.46%, and 63.43 ± 2.25% at baseline, 1 and 2 years, respectively. In this group, the repeated measures ANOVA test showed *p* = 0.636. The mean SFCT of MacTel group were 324.65 ± 89.65μm, 326.14 ± 93.11μm, and 322.65 ± 91.77μm at baseline, 1 and 2 years, respectively (*p* = 0.436), whereas the mean SFCT of control group were 304.30 ± 51.86 μm, 300.86 ± 52.64μm, and 298.55 ± 53.71μm at baseline, 1 and 2 years, respectively (*p* = 0.275).

**Table 2:** Choroidal vascularity index (%) and subfoveal choroidal thickness changes over time.

	Baseline	1 year	2 years	*p*-value
MacTel CVI(n = 43)	64.59 ± 2.92	63.76 ± 2.67	62.97 ± 2.74	0.001
Control CVI(n = 30)	63.33 ± 2.45	63.04 ± 2.46	63.43 ± 2.25	0.636
MacTel SFCT(n = 43)	324.65 ± 89.65	326.14 ± 93.11	322.65 ± 91.77	0.436
Control SFCT(n = 30)	304.30 ± 51.86	300.86 ± 52.64	298.55 ± 53.71	0.275

Data are expressed as mean ± standard deviation (95% confidence interval). Repeated measures ANOVA tests.

Abbreviations: CVI, Choroidal vascularity index; MacTel, macular telangiectasia; SFCT, subfoveal choroidal thickness.

## Discussion

In our study, we analyzed the choroidal structures in MacTel type 2 by measuring SFCT and CVI with SS-OCT images. First, we compared SFCT and CVI between MacTel type 2 group and the sex and age matched control group at baseline. Previous studies suggested the choroidal involvement in the disease process since retinal-choroidal anastomoses are observed in MacTel type 2 [8,9]. However, studies showed a disagreement on the CT analysis in the disease. Nunes et al. [11] presented a greater mean CT measurement in MacTel type 2 compared to control, whereas Wang et al. [10] and Chhablani et al. [12] showed that MacTel type 2 had no significantly different CT than control eyes. Our study confirmed the latter study results, where no SFCT difference was seen in MacTel type 2 eyes compared to control eyes at baseline.

Also, Wang et al. [10] analyzed the choroidal vascular structure changes in MacTel type 2 using CVD by binarizing *en face* SS-OCT images, and reported that no significant CVD difference was seen in the disease compared to controls. Our study evaluated the changes in choroidal vascular structure by measuring CVI instead of CVD in MacTel Type 2, and we came with a similar result, that CVI of the disease was not significantly different from the sex and age-matched control group at baseline.

In addition to comparing the CT and CVI in MacTel type 2 and control group at baseline, we also analyzed their changes over time. In case of SFCT, neither MacTel type 2 nor control group showed significant changes over time on a yearly basis. Similarly, when we measured the CVI of healthy controls on a yearly basis, there was no statistically significant changes. It is previously reported that CVI changes significantly with age as its decrease was notable among each decade age group from the 4^th^ decade of life [18]. The disagreement on the CVI changes with ages could be due to the different measuring period, since our study evaluated yearly changes of CVI during 2 years. On the contrary, our study showed that MacTel type 2 presented a significant decrease in CVI over time from baseline to 2 years when yearly measured.

Although it is widely known through many studies that Müller cell dysfunction or alteration is highly responsible for the disease progress [6,7], the exact pathogenesis of the disease has not yet been discovered. The progressive CVI reduction in MacTel type 2 strengthens the idea of choroidal involvement in the disease. There have been studies where CVI reduction was seen in exudative age-related macular degeneration (AMD) and polypoidal choroidal vasculopathy (PCV) [15,19,20]. Also, Kim at el. [21] proposed that central serous chorioretinopathy (CSC) had a statistically lower CVI when choroidal neovascularization(CNV) was accompanied. They suggested secondary choroidal changes due to CNV or underlying choroidal ischemia associated with the development of CNV [21]. However, none of our study patients had CNV but still showed a decreasing trend in choroidal vascular structures. This result may suggest that the reduction of choroidal vascular structure, measured using CVI, could be either a cause or a result of progression of MacTel type 2 itself without CNV. Of course, a cause and effect relationship cannot be determined without pathologic confirmation. In addition, this reduction in CVI may represent a tendency toward choroidal ischemia as the disease progresses, which possibly contributes to development of CNV.

Since Müller cell dysfunction is also involved in the MacTel type 2 as mentioned above, it would be of interest to evaluate the relationship between Müller cell and choroid. In addition, the choroidal vascular structure of MacTel type 2 with CNV could be investigated as a further study to confirm their actual relationship.

There were some limitations of the current study. First, the number of eyes included in the study was relatively small. Second, only subfoveal region of 1500 μm (750 μm either side of the fovea) was used to measure CVI since the disease mainly affect the subfoveal area. There is a possibility that the result may have been different if larger choroidal area was included in the measurement. Third, due to the limited number of study patients, the stages of MacTel type 2 could not be classified and analyzed separately, which demands further studies. Forth, CVI was measured from a single two-dimensional scan, which may not be able to represent the entire choroidal vascular structure. Lastly, due to the retrospective nature of the study, the time at which the OCT images were acquired could not be controlled. Diurnal variation of CT was previously reported [22], and it might have influenced our study results although the diurnal variation of CVI has not yet been demonstrated. However, to the best of our knowledge, this is the first report showing choroidal structural changes in MacTel type 2 using the CVI.

In conclusion, CVI decreases at a faster rate in MacTel type 2 in comparison with sex and age-matched healthy subjects although their baseline CVI was not significantly different from each other. This may suggest possible choroidal involvement in the progression of MacTel type 2 and the cause and effect relationship may be confirmed through further studies. Also, it would be valuable to further investigate the relationship between choroid and Müller cell.

## Supporting information

S1 File Dataset. (XLSX)
